# An investigation of the constancy of effect in Cochrane systematic reviews in context with the assumptions for noninferiority trials

**DOI:** 10.1186/s12874-022-01684-9

**Published:** 2022-07-25

**Authors:** Enass M. Duro, Steven A. Julious, Shijie Ren

**Affiliations:** School of Health and Related Research (ScHARR), The University of Sheffield, Sheffield, UK

**Keywords:** Noninferiority trial, Constancy assumption, Indirect comparison, Cochrane reviews

## Abstract

When designing a noninferiority (NI) study one of the most important steps is to set the noninferiority (NI) limit. The NI limit is an acceptable loss of efficacy for a new investigative treatment compared to an active control treatment – often standard care. The limit should be a value so small that the loss efficacy is clinically zero. An approach to the setting of a noninferiority limit such that an effect over placebo can be shown through an indirect comparison to placebo-controlled trials where the active control treatment was compared to placebo. In this context, the setting of the NI limit depends on three assumptions: assay sensitivity, bias minimisation, and the constancy assumption. The last assumption of constancy assumes the effect of the active control over placebo is constant. This paper aims to assess the constancy assumption in placebo-controlled trials.

**Methods:** 236 Cochrane reviews of placebo-controlled trials published in 2015–2016 were collected and used to assess the relation between the placebo, active treatment, and the standardised treatment different (SMD) with the time (year of publication).

**Results: **The analysis showed that both the size of the study and the treatment effect were associated with year of publication. The three main variables that affect the estimate of any future trial are the estimate from the meta-analysis of previous trials prior to the trial, the year difference in the meta-analysis, and the year of the trial conduction. The regression analysis showed that an increase of one unit in the point estimate of the historical meta-analysis would lead to an increase in the predicted estimate of future trial on the SMD scale by 0.88. This result suggests the final trial results are 12% smaller than that from the meta-analysis of trials until that point.

**Conclusion: **The result of this study indicates that assuming constancy of the treatment difference between the active control and placebo can be questioned. It is therefore important to consider the effect of time in estimating the treatment response if indirect comparisons are being used as the basis of a NI limit.

**Supplementary Information:**

The online version contains supplementary material available at 10.1186/s12874-022-01684-9.

## Introduction

Noninferiority (NI) trials have an important place in clinical trial design and analysis and often they can be the only opportunity to answer certain clinical questions [1]. There are a number of reasons for choosing a NI trial over of a superiority design including a wish to investigate whether the test treatment tolerated better by patients than the active control; has a better safety profile or is less expensive. For these reasons, equal or less efficacy of the test compared to active-control could be acceptable [2].

When designing a noninferiority study one of the most important steps is to set the noninferiority (NI) limit. The NI limit is a defined as an acceptable loss of efficacy for a new investigative treatment compared to an active control treatment often standard care. The limit should be a value so small the loss efficacy should be clinically zero [3].

An approach to the setting of the NI limit is to set it at a level such than an effect for the new investigative treatment could be shown over placebo through an indirect comparison to placebo-controlled trials where the active control treatment was compared to placebo. In this context, the setting of the NI margin depends on the ABC assumptions of [4]:

**A**ssay sensitivity – is the current study able to show an effect for the control treatment. This can be an issue for example if the trial procedures are optimised for the investigative treatment meaning the effect for the control treatment is smaller than previously observed.

**B**ias minimisation – is the study well designed with randomisation and level of blinding.

**C**onstancy – is the effect of the control treatment over placebo constant over time.

The assumption of constancy is the assumption which will be investigated in this paper. Studies have shown an improvement of the placebo response over time in different therapeutic areas[5–7]. This in has turn led to a reduction in the effect observed for the comparator treatment and so lack of constancy. The impact for the setting of a NI limit is that if studies are designed under the assumption of constancy their estimate of effect will be biased and overstated [8].

## Aim and objectives

The paper investigated if the effect of the placebo and active control change over time using the Cochrane reviews of placebo-controlled trials published in 2015/2016.

In the paper:

- The effect of year of publication (as a proxy to the time of trial conduction) on the difference between the active treatment and the placebo (effect size) over time is assessed.
- Factors that affect the estimate of a future trial based on the available historical trials using the weighted linear regression are investigated.

## Methods

### Study design and data collection

A search for the Cochrane systematic reviews of placebo-controlled trials published in the Cochrane database from January 2015 to December 2016 was undertaken. This research was started in 2017 and because of that 2015, 2016 were chosen as the most recent years.

### Inclusion criteria for the systematic review

The inclusion criteria for selecting the relevant systematic reviews were:

- 1. Cochrane reviews of placebo-controlled trials
- 2. Defined as placebo-controlled trials by the review’s author regardless of the type of control group used (placebo, no treatment, and usual care).
- 3. Meta-analysis was performed.
- 4. The meta-analysis included at least four placebo-controlled trials (4 trials was used to ensure that the SMD that measure after deleting the last trial is measure from at least 3 trials to reduce the chance of extreme results).

- 5. Meta-analyses published in 2015–2016

The exclusion criteria were:

- 1. Reviews that were withdrawn from publication
- 2. Over reviews or reviews that included active-controlled trials
- 3. Reviews containing three or fewer trials
- 4. Reviews where meta-analysis was not performed
- 5. Reviews where all trials were conducted in the same year

To build the weighted regression model, a database that contains the original estimate of the treatment effect from the meta-analysis and 95% confidence interval (CI) and the significance level, the standardised mean difference (SMD) and its 95% CI for all trials in the meta-analysis, the calculated SMD after deleting the last trial(s) and it 95% CI, and the SMD for the last trial (s) was formulated. It also includes the number of trials included in each meta-analysis, year difference between the last trials and first trials, and year difference between last trial and most recent trial after deleting the last trial. The unit of analysis is the overall meta-analysis result not the individual trial results.

For each meta-analysis in the formulated database, three standardised mean differences (SMD) were calculated:

- The SMD for all trials included in the original meta-analysis: SMD
- The SMD for all trials included in the original meta-analysis excluding the most recent (last) trial(s): SMDdl
- The SMD for the most recent (last) trial(s):SMDlt

### Statistical analysis

#### Correlation between the SMD and time

A correlation coefficient was used in previous studies to measure the association between the year of publication and the effect size [9]. In this analysis, the correlations—both for all trials in general and by the meta-analysis – between the year of publication and: the SMD; placebo effect and active treatment and sample size were estimated. The year of publication was used as a proxy for the year of trial: this information was available for all trials and has been used in before as a proxy for year of conducting the trial [6, 7, 9, 10].

The reviews included both reviews with positive (for example, healing, improvement) and adverse outcomes (for example, death, relapse, pain intensity). To perform one scale of measure, the SMD for the reviews of negative outcomes was transformed into a positive outcome, and then the correlations were calculated.

The correlation is considered weak if the correlation coefficient is from [0, 0.3], moderate if the correlation coefficient is [0.3, 0.5], and a correlation coefficient of more than 0.5 is considered a strong correlation [11]. The aim of subdividing the correlations into weak, moderate, and strong correlations was to demonstrate the percentage of correlations that fell into these three categories regardless of the sign of the correlation.

### Building the weighted regression model:

The main aim was to investigate the factors that affect the estimate from previous trials and if it is possible to predict the estimate of a trial based on a meta-analysis of previous similar trials using a regression model. Due to the nature of the outcome variable available from the constructed data set, a multiple weighted linear regression model will be the appropriate model to use to construct the predictive model.

A model was developed to predict the values of SMDlt of the predicted trial (dependent variable), using the (SMDdl) from the meta-analysis of historical trials as the independent variable and the year of the predicted trial, the year differences between the oldest and latest trial in the meta-analysis of historical trials and the year of the predicted trial were tested as possible co-variables in the model.

The independent variable (SMDdl) used in the model was constructed from a meta-analysis of several trials, and because of that, each case in the data set will have a different weight in according to the sample size of the meta-analysis. For this reason the regression model was weighted multiple regression (WLS) [12]. The model was weighted by the total sample size of the historical meta-analysis.

Analyses were undertaken in SPSS [13] and R [14].

## Results

### Data extraction

**Fig. 1:**
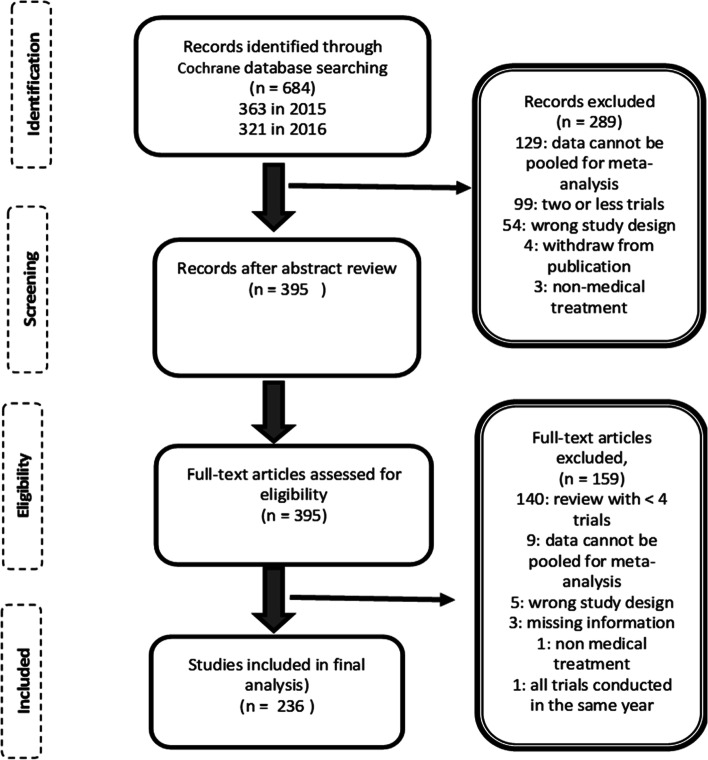
Flowchart for the process of data extraction

From the Cochrane database, 684 titles were identified to have a placebo term in the abstract or the title. Of these, 289 titles were excluded after reviewing the abstract, and 98 titles were excluded after a secondary assessment of the review (reviewing the manuscript). The final sample included 236 reviews for analysis. Figure 1 represents the flow diagram for the data extraction process. The process of data extraction and reviewing the abstract was done by the main author (Duro). To control for any biases, the search was done twice, and another data set was formulated. Finally, both data sets were compared for any mismatch. All data extraction was done under supervision of the second and third authors.

The main reasons for exclusion in the full-text article assessment were three or fewer trials in the review (238 reviews): 53 reviews had no trials; 56 reviews had one trial; 69 had only two trials in the review; and 60 reviews had three trials. In 138 reviews, data could not be pooled for meta-analysis.

Additionally, 59 reviews had the wrong study design: 52 were for active-controlled trials; two reviews were network meta-analysis; and five reviews were overviews of Cochrane reviews. Four reviews used non-medical treatment; four reviews were withdrawn from publication; three reviews had missing information; and in one review, all trials were conducted in the same year.

In total, 2489 placebo-controlled trials from 236 meta-analyses were included in the final analysis. Among the meta-analyses, 155 (65.4%) measured negative outcomes, and 82 (34.6%) measured positive outcomes. The median number of trials was seven trials, and the mean was 9.9 trials, with a minimum number of four trials and a maximum of 51 trials.

The years of trial conducting ranged from 1931 to 2016. The year difference ranged from one year to 80 years. Among the included meta-analyses, 76 (32.1%) used mean difference as the measure of effect. The risk ratio was used in 131 (55.3%), the odds ratio in 27 (11.4%) of the meta-analyses, and the risk difference in only three meta-analyses (1.2%). The most common outcome measured was pain, 30 (12.17%), followed by death, 26 (11%), in the included meta-analyses. The median sample size was 1160 participants with IQR (interquartile range) (494—2229), the minimum sample size was 105 and the maximum was 43,290 participants.

Table 1 gives the different Cochrane groups included in the review. There were 17 different therapeutic areas; the most frequently occurring was Gynaecology and Obstetrics with 37 (15.6%) reviews.

**Table 1:** Distribution of the reviews by Cochrane groups

Cochrane group	Frequency
Pain, Palliative and Supportive Care Group	22.0 (9.3%)
Pregnancy and Childbirth Group	20.0 (8.5%)
Gynaecology and fertility group	15.0 (6.4%)
Heart Group	13.0 (5.5%)
Anaesthesia, Critical and Emergency Care Group	11.0 (4.7%)
IBD Group	11.0 (4.7%)
Musculoskeletal Group	10.0 (4.2%)
Stroke Group	9.0 (3.8%)
Kidney and Transplant Group	9.0 (3.8%)
Airway group	8.0 (3.4%)
Hypertension Group	8.0 (3.4%)
Acute Respiratory Infections Group	7.0 (3.0%)
Infectious Disease Group	7.0 (3.0%)
Vascular Group	7.0 (3.0%)
Common Mental Disorders Group	6.0 (2.5%)
Drugs and Alcohol Group	5.0 (2.1%)
ENT Group	5.0 (2.1%)
Neonatal Group	5.0 (2.1%)
Neuromuscular group	5.0 (2.1%)
Schizophrenia Group	5.0 (2.1%)
Skin Group	5.0 (2.1%)
Upper GI and Pancreatic Diseases Group	5.0 (2.1%)
Developmental, Psychosocial and Learning Problems Group	3.0 (1.3%)
Epilepsy Group	3.0 (1.3%)
Wounds Group	3.0 (1.3%)
Hepato-Biliary Group	3.0 (1.3%)
Tobacco Addiction Group	3.0 (1.3%)
Bone, Joint and Muscle Trauma Group	2.0 (0.8%)
Cystic Fibrosis and Genetic Disorders Group	2.0 (0.8%)
Dementia and Cognitive Improvement Group	2.0(0.8%)
Eye and Vision Group	2.0 (0.8%)
Haematological Malignancies Group	2.0 (0.8%)
Incontinence Group	2.0 (0.8%)
Metabolic and Endocrine Disorders Group	2.0 (0.8%)
Movement Disorders Group	2.0 (0.8%)
Other groups	7.0 (3.0%)
Total	236.0 (100.0%)

*IBD* inflammatory bowel disease

### Results of Correlations

Correlations between the year of publication and sample size, placebo effect, active treatment effect, SMD were obtained. Both Pearson and Spearman correlations were estimated. Correlations were measured for the 2489 trials in general and then individually for each meta-analysis. The results for parametric and non-parametric correlations were similar.

For all included trials the correlation between the sample size of the trial and the year of publication was positively correlated with the Pearson’s correlation of 0.038, [95% CI: 0.006; 0.086]. Year of publication was negatively correlated with SMD, with Pearson’s correlation of—0.013 [-0.055; 0.03].

58.5% of the reviews had a negative correlation between the SMD and the year of publication. The median correlation was—0.12, mean was -0.083 [95% CI -0.138: -0.028]. In 58.6% of the reviews there was a positive correlation between the placebo response and the year of publication (Table 2). The median correlation was 0.09, mean was 0.07, 95% CI [0.0126, 0.127]. In 51.3% of the reviews had a negative correlation between the active treatment response and the year of publication (Table 2). The median correlation was—0.04, mean 0.02, 95% CI [-0.0749, 0.0349] (Table 2).

**Table 2:** Correlation between SMD, the placebo response and the active treatment response and the year of publication

Correlation	SMD	Placebo, N (%)	Active treatment, N (%)
Strong Negative	47 (19.9%)	29 (12.9%)	34 (15.1%)
Moderate Negative	38 (16.1%)	16 (7.1%)	31 (13.8%)
Weak Negative	53 (22.5%)	47 (20.9%)	51 (22.7%)
Weak Positive	45 (19.1%)	60 (26.7%)	58 (25.8%)
Moderate Positive	32 (13.6%)	35 (15.6%)	18 (8%)
Strong Positive	21 (8.9%)	38 (16.9%)	33 (14.7%)
Total	236. (100%)	226 (100%)	226 (100%)

*SMD* Standardised Mean Difference*, N* (total number of reviews)

### Building the regression model

A weighted multiple regression model was undertaken to test if the SMD of the last trial (SMDlt) can be predicted from SMD of previous meta-analysis (SMDdl) and what are the changes in SMDdl. The model includes SMD of the last trial as the dependent variable (SMDlt), SMD from the previous meta-analysis as the independent (predictor) variable (SMDdl).

The covariates tested in the model were: the year difference between the latest trial and the oldest trial in the meta-analysis of historical trials, the year of publication of predicted trial. The model was weighted by the sample size of the historical meta-analysis. Stepwise regression was used. Model Assumptions were checked (Supplementary materials).

Table 3 illustrates the results of the weighted regression model.

**Table 3:** Summary of the results of the regression model

	B	95%CI	*P* value
(Constant)	36.14	[15.20; 57.08]	0.001
SMDdl	0.88	[0.76; 1.01]	< 0.001
Year difference	-0.009	[-0.013; -0.004]	< 0.001
Year of future trial	-0.018	[-0.028; -0.007]	0.001

Weighted Least Squares Regression—Weighted by sample size of the historical meta-analysis

Dependent Variable: Standardized mean difference future trial, Year difference = year difference between latest trial and the oldest trial, SMDdl = Standardized mean difference of historical trials

The final fitted regression model is.

*y (SMD future trial)* = *36.14* + *0.88* × *(SMD of historical trials)—0.009(Year difference between oldest and latest trial in the historical meta-analysis)—0.018 (year of publication of the future trial).*

The results of final regression model include 224 reviews. The model indicated that SMD from the meta-analysis of historical trials (SMDdl), Year difference and the year of future trial explain 50% of the variance in the model ($\mathrm{Adjusted} {R}^{2}$= 0.50). SMD of the historical meta-analysis statistical significantly predict the SMD of future trial (β = 0.88, 95%CI [0.76; 1.01]). This result infers that for each unit increase in SMD of an historical meta-analysis the SMD of the future trial will increase by 0.88 unit.

For the year difference between the oldest trial and the latest trial for every one-year increase in the difference the SMD of the future trial will decrease by -0.009, 95%CI [-0.013, 0.004] The year of the future trial was statistical significantly predict the SMD of the future trial by—0.018, 95%CI [ -0.028, -0.007], For each year increase in the future trial, SMD will be reduced by 0.018.

The full model’s selection and summary provided in the supplementary material.

## Discussion

The aim of this study was to investigate the changes of the placebo and active treatment over time by measuring the correlation between the SMD and the year of publication and by assess the degree of the relation using the regression model. The results from this study indicate that in placebo-controlled trials the difference between the placebo and active treatment is not constant over time. There is an argument that the improvement in the placebo effect group is due to changes in the population and the standard treatment [15]. In the analysis in the paper there were also changes in the sample size over time. These results infer a regression to the mean effect as large studies were undertaken later in the meta-analysis.

To predict the effect size of future trial a regression model was built. The three main variables that affect the estimate of any future were the estimate from the meta-analysis of historical trials, the year difference in the meta-analysis and the year of the predicted trial. Increasing of one unit in the point estimate of the historical meta-analysis will lead to an increase in the predicted estimate of future trial by 0.88. This result suggests the final trial results are 12% smaller than that from the meta-analysis of trials until that point.

With NI trials the estimate from the historical placebo-controlled trials can be used estimate of the treatment difference between the putative placebo and the active control in NI trial. However, the results in this paper show estimate could be biased estimate and does not reflect the actual efficacy of the active control compared to the putative placebo.

the results are in line with the paper of Ioannidis et al.[5] that concludes the treatment effect is not constant over time and highlight an important issue of the bias that could arise from using the estimate of historical meta-analysis for indirect comparison without any further adjustment.

This paper is also consistent with findings for the situation when two clinical trials are done in sequence, but the second trial only starts conditional on the result of the first trial. Here the first study will report effects larger than the second study [16]. If the first study had 80% power and need to be statistically significant for the second study to start, it will have effects 11% bigger the second study [16].

The results in the paper and that from the literature highlights the need to use the most appropriate estimate of effect. This could be from most recent trials as opposed to an overall effect across trials [17]. The use of network meta-analysis (NetMAP) instead of pair wise meta-analysis and comparing the placebo indirectly with active treatment could be the answer to this issue [2].

A limitation of the paper is that, only published data were used. For this review, 72% of the included meta-analyses had statistically significant results. This could increase the possibility of publication bias. However, it should be noted that this method of data analysis could be used in the indirect comparison situations to estimation of the NI margin from the historical data. A further limitation is the use of the year of publication as a surrogate for the year of trial conducting.

## Conclusion

The result of this study indicates that assuming constancy of the treatment difference between the active control and placebo can be questioned. It is therefore important to consider the effect of time in estimating the treatment response if indirect comparisons are being used as the basis of a NI limit.

## Supplementary Information

**Additional file 1.** Supplementarymaterial.

## Data Availability

The datasets generated and/or analysed during the current study are not publicly available because it is a part form PhD thesis and needed for other unpublished work. but are available from the corresponding author on reasonable request.

## Abbreviations

CI: Confidence Interval
NI: Non-inferiority trial
SMD: Standardised Mean Difference
SMDdl: Standardised Mean Difference deleting last trial
SMDlt: Standardised Mean Difference for the latest trial
