# Supplementary material for: An investigation of the constancy of effect in Cochrane systematic reviews in context with the assumptions for noninferiority trials
Source: BMC Med Res Methodol. 2022 Jul 25;22:204. doi: 10.1186/s12874-022-01684-9 (PMC9316704; doi:10.1186/s12874-022-01684-9)
Supplement: Supplementary file 1 — Additional file 1. Supplementarymaterial. [file 12874_2022_1684_MOESM1_ESM.docx]

# Supplementary material

## Inclusion Criteria for the Systematic Review

The inclusion criteria for selecting the relevant systematic reviews were:

1. Cochrane reviews of placebo-controlled trials
2. Defined as placebo-controlled trials by the review’s author regardless of the type of control group used (placebo, no treatment, and usual care).
3. Meta-analysis was performed.
4. The meta-analysis included at least four placebo-controlled trials (4 trials was used in ensure that the SMD that measure after deleting the last trial is measure from at least 3 trials in reduce the chance of extreme results).
5. Meta-analyses published in 2015-2016

The exclusion criteria were:

1. Reviews that were withdrawn from publication
2. Over reviews or reviews that included active-controlled trials
3. Reviews containing three or fewer trials
4. Reviews where meta-analysis was not performed
5. Reviews where all trials were conducted in the same year

Rayyan (18), a web-based systematic review manager, was used to screen the systematic reviews for inclusion and retrieve the needed information. The keyword used in the primary search in the title and abstract was “placebo”, the abstracts were reviewed, and the inclusion and the exclusion criteria were applied.

from each review included in the study, information regarding Cochrane ID, publication year, Cochrane group, and medical speciality was retrieved. From each review, the first meta‑analysis in the report was chosen for the analysis unless it had no meta-analysis or had less than four trials. If the first meta-analysis could not be chosen, the next meta-analysis with more trials was chosen. If in any meta-analysis, the subgroups shared the weight in the study, they were included as one analysis. If the weight of the study was not shared between the subgroups, only the subgroup with the largest number of trials was included.

From each included meta-analysis, information regarding: the year of publication; number of trials included type of control group used; the active treatment used; total number of patients; weight of each trial; total number of patients in the placebo and active treatment group; placebo and active treatment effect; measure of effect used; type of analysis (fixed or random) and heterogeneity were retrieved. Besides, the main estimate and 95% CI information regarding the risk of bias and the quality of evidence were collected.

## Standardising the difference

There was a need to obtain a standardised measure of effect to compare both the binary data and numerical data. For the binary data, the measure of effect was transferred to the odds ratio and then the standardised mean difference (SMD). The SMD was calculated from the odds ratio (19) using the equations below

$SMD=Log OddsRatio\times\frac{\sqrt{3}}{\pi}$, (1)

$V_{smd}=V_{logoddsratio}\times\frac{3}{\pi^{2}}$, (2)

$V_{logoddsratio}=\frac{1}{A}+\frac{1}{B}+\frac{1}{C}+\frac{1}{D}$, (3)

where V denotes the variance of the log odds ratio, A is the number of events in the treatment group, B is the number of no events in the treatment group, C is the number of events in the control group and D is the number of no events in the control group.

For continuous data, the measure of effect was the mean difference that was transformed into the SMD

$SMD=\frac{\bar{X1}-\bar{X2}}{S_{within}}$, (4)

$S_{within}=\sqrt{\frac{\left( n_{1}-1 \right)S_{1}^{2}+\left( n_{2}-1 \right)S_{2}^{2}}{n_{1}+n_{2}-2}},$ (5)

$V_{smd}=\frac{n_{1}+n_{2}}{n_{1}n_{2}}+\frac{d^{2}}{2\left( n_{1}+n_{2} \right)}.$ (6)

Here $S_{within}$ is the within-groups standard deviation, pooled across groups, S_1_ is the standard deviation of the placebo group, S_2_ is the standard deviation of the control group, $n_{1} is the sample size of the control group$, $n_{2}$ is the sample size of the active treatment group, and $V_{\mathrm{smd}} is the variance of the SMD$.

## Checking the regression model adequacy


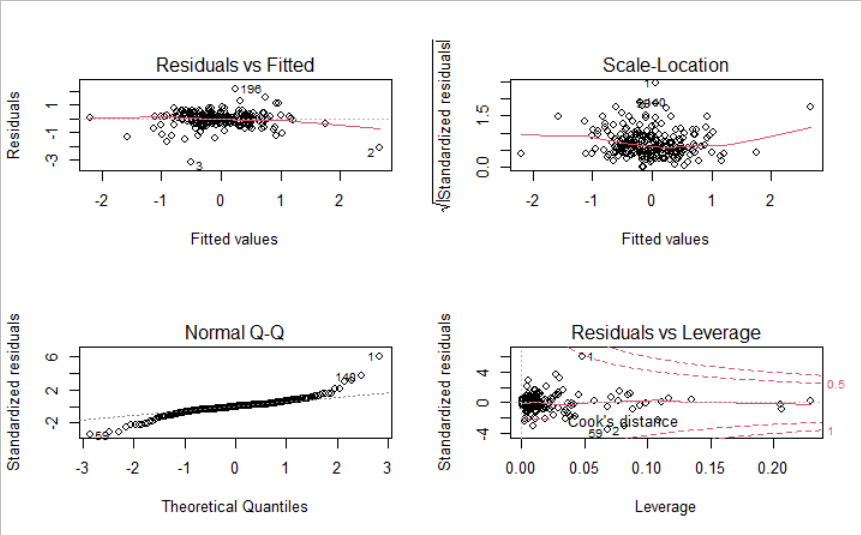


Figure 1: checking the assumptions of the regression model

| *Table1:Models Summary* | | | | | | | | |
| --- | --- | --- | --- | --- | --- | --- | --- | --- |
| Model | | Unstandardized Coefficients | | Standardized Coefficients | t | Sig. | 95.0% Confidence Interval for B | |
|  |  | B | Std. Error | Beta |  |  | Lower Bound | Upper Bound |
| 1 | (Constant) | -.028 | .028 |  | -1.016 | .311 | -.083 | .026 |
|  | SMDdl | .852 | .065 | .662 | 13.153 | .000 | .725 | .980 |
| 2 | (Constant) | .145 | .052 |  | 2.774 | .006 | .042 | .249 |
|  | SMDdl | .867 | .063 | .673 | 13.770 | .000 | .743 | .992 |
|  | year difference in the meta-analysis | -.008 | .002 | -.189 | -3.857 | .000 | -.013 | -.004 |
| 3 | (Constant) | 36.144 | 10.625 |  | 3.402 | .001 | 15.204 | 57.083 |
|  | SMDdl | .887 | .062 | .689 | 14.349 | .000 | .765 | 1.009 |
|  | year difference in the meta-analysis | -.009 | .002 | -.192 | -4.026 | .000 | -.013 | -.004 |
|  | Last year of publication | -.018 | .005 | -.162 | -3.388 | .001 | -.028 | -.007 |
| a. Dependent Variable: SMDlt | | | | | | | | |
| b. Weighted Least Squares Regression - Weighted by Ndl | | | | | | | | |

| *Table 2: Changes in R Square* | | | | | | | | | |
| --- | --- | --- | --- | --- | --- | --- | --- | --- | --- |
| Model | R | R Square | Adjusted R Square | Std. Error of the Estimate | Change Statistics | | | | |
|  |  |  |  |  | R Square Change | F Change | df1 | df2 | Sig. F Change |
| 1 | .662^a^ | .438 | .435 | 20.11362 | .438 | 172.990 | 1 | 222 | .000 |
| 2 | .688^b^ | .473 | .469 | 19.51298 | .035 | 14.877 | 1 | 221 | .000 |
| 3 | .707^c^ | .500 | .493 | 19.06616 | .026 | 11.480 | 1 | 220 | .001 |
| a. Predictors: (Constant), SMDdl | | | | | | | | | |
| b. Predictors: (Constant), SMDdl, year difference in the meta-analysis | | | | | | | | | |
| c. Predictors: (Constant), SMDdl, year difference in the meta-analysis, Last year of publication | | | | | | | | | |
